# Insights into the Cnx1E catalyzed MPT-AMP hydrolysis

**DOI:** 10.1042/BSR20191806

**Published:** 2020-01-10

**Authors:** Thomas W. Hercher, Joern Krausze, Sven Hoffmeister, Dagmar Zwerschke, Thomas Lindel, Wulf Blankenfeldt, Ralf R. Mendel, Tobias Kruse

**Affiliations:** 1 TU Braunschweig, Institute of Plant Biology, Spielmannstrasse 7, 38106 Braunschweig, Germany; 2 TU Braunschweig, Institute of Organic Chemistry, Hagenring 30, 38106 Braunschweig, Germany; 3 Structure and Function of Proteins, Helmholtz Centre for Infection Research, Inhoffenstrasse 7, 38124 Braunschweig, Germany; 4 TU Braunschweig, Department for Biotechnology, Institute of Biochemistry, Biotechnology and Bioinformatics, Spielmannstrasse 7, 38106 Braunschweig, Germany

**Keywords:** Adenylated molybdopterin, Enzyme catalyzed hydrolysis, Molybdenum cofactor, Molybdenum insertase

## Abstract

Molybdenum insertases (Mo-insertases) catalyze the final step of molybdenum cofactor (Moco) biosynthesis, an evolutionary old and highly conserved multi-step pathway. In the first step of the pathway, GTP serves as substrate for the formation of cyclic pyranopterin monophosphate, which is subsequently converted into molybdopterin (MPT) in the second pathway step. In the following synthesis steps, MPT is adenylated yielding MPT-AMP that is subsequently used as substrate for enzyme catalyzed molybdate insertion. Molybdate insertion and MPT-AMP hydrolysis are catalyzed by the Mo-insertase E-domain. Earlier work reported a highly conserved aspartate residue to be essential for Mo-insertase functionality. In this work, we confirmed the mechanistic relevance of this residue for the *Arabidopsis thaliana* Mo-insertase Cnx1E. We found that the conservative substitution of Cnx1E residue Asp274 by Glu (D274E) leads to an arrest of MPT-AMP hydrolysis and hence to the accumulation of MPT-AMP. We further showed that the MPT-AMP accumulation goes in hand with the accumulation of molybdate. By crystallization and structure determination of the Cnx1E variant D274E, we identified the potential reason for the missing hydrolysis activity in the disorder of the region spanning amino acids 269 to 274. We reasoned that this is caused by the inability of a glutamate in position 274 to coordinate the octahedral Mg^2+^-water complex in the Cnx1E active site.

## Introduction

The molybdenum cofactor (Moco) biosynthesis pathway involves the concerted action of numerous enzymes that are conserved throughout all kingdoms of life [1,2]. The initial substrate for Moco biosynthesis is GTP, which is converted into cyclic pyranopterin monophosphate (cPMP) in the first reaction of a multi-step pathway [2]. This reaction involves radical S-adenosyl methionine chemistry and is catalyzed by the cPMP synthase [3]. In the following step of Moco biosynthesis, cPMP is converted into molybdopterin (MPT), a reaction that is distinguished by the introduction of the dithiolene motif characteristic for MPT. This reaction is catalyzed by the heterotetrameric MPT-synthase complex, which comprises two large and two small subunits [4,5]. Here, the two large subunits form the core of the complex, while each of the two small subunits interacts independently with one of the large subunits [5]. Molybdate is inserted into the MPT backbone in a subsequent step, yielding Moco. *In vivo* interaction studies revealed the plant MPT synthase complex and the molybdenum insertase (Mo-insertase) to interact with each other, thus providing the framework necessary for efficient, protected and directed metabolite transfer [6]. The molybdate insertion reaction involves both functional domains of Mo-insertases, namely E- and G-domain [7] whose role for the Mo-insertion reaction has been studied in detail using the plant (*Arabidopsis thaliana*) Mo-insertase Cnx1 as model enzyme [8–15]. Notably, these domains are reactive as separately expressed domains (prokaryotes) or fused together (eukaryotes, except the lower alga *Chlamydomonas reinhardtii* [16]). Initially, the Mo-insertase G-domain catalyzes the ligation of an AMP molecule to the terminal phosphate group of MPT, yielding adenylated MPT (MPT-AMP, [11,12]). Subsequently, MPT-AMP is transferred to the Mo-insertase E-domain, which requires molybdate to be bound to the E-domain oxo-anion entry site [15]. Within the Cnx1E active site, MPT-AMP adopts a conformation that is different from the one found in the Cnx1G MPT-AMP co-structure [11] and that properly orients the dithiolene motif toward enzyme bound molybdate [14,15]. Subsequently the MPT-AMP phosphoric anhydride bond is hydrolyzed, a reaction that is believed to be the prerequisite for enzyme catalyzed molybdate insertion into the MPT dithiolene motif [13–15]. Recent work suggested that the Cnx1E catalyzed molybdate insertion reaction involves the relocation of molybdate from the initial oxo-anion binding site to the insertion site [15]. Upon synthesis Moco is transferred to the cellular user enzymes and/or to the cellular Moco transfer/storage system [2,6,17,18]. Within this work we describe the identification and, for the first time, the biochemical characterization of a hydrolysis inactive Cnx1E variant (Cnx1E D274E) that may pave the way to decipher the molecular mechanism(s) underlying Cnx1E reactivity.

## Materials and methods

### Generation of Cnx1E variant D274E

Cnx1E variant D274E was generated following the QuikChange (Agilent Technologies) protocol modified for the use of Phusion® High-Fidelity DNA Polymerase (Thermo Fischer). As described earlier [15], we used the Cnx1E wild-type pGPlus expression vector [14] as template for PCR-based mutagenesis. The sequence of the primer pair used to introduce the single amino acid exchange D274E was 5´-gggagacagggaGttcgtcaagccattactcgaag-3´ and 5´-agtaatggcttgacgaaCtccctgtctcccattga-3´ (mismatches in upper case). The identity of the generated construct was subsequently confirmed by sequencing.

### Expression and purification of recombinant Cnx1E

For crystallization experiments, recombinant Cnx1E was expressed and purified as described previously [15]. For recombinant biochemistry, Cnx1E was expressed and purified as described previously [14].

### *In vitro* transfer of MPT-AMP on Cnx1E

*In vitro* transfer of MPT-AMP on Cnx1E was essentially carried out as described previously [14].

### HPLC-based quantification of Cnx1E bound MPT-AMP and Moco/MPT

**Figure 1:**
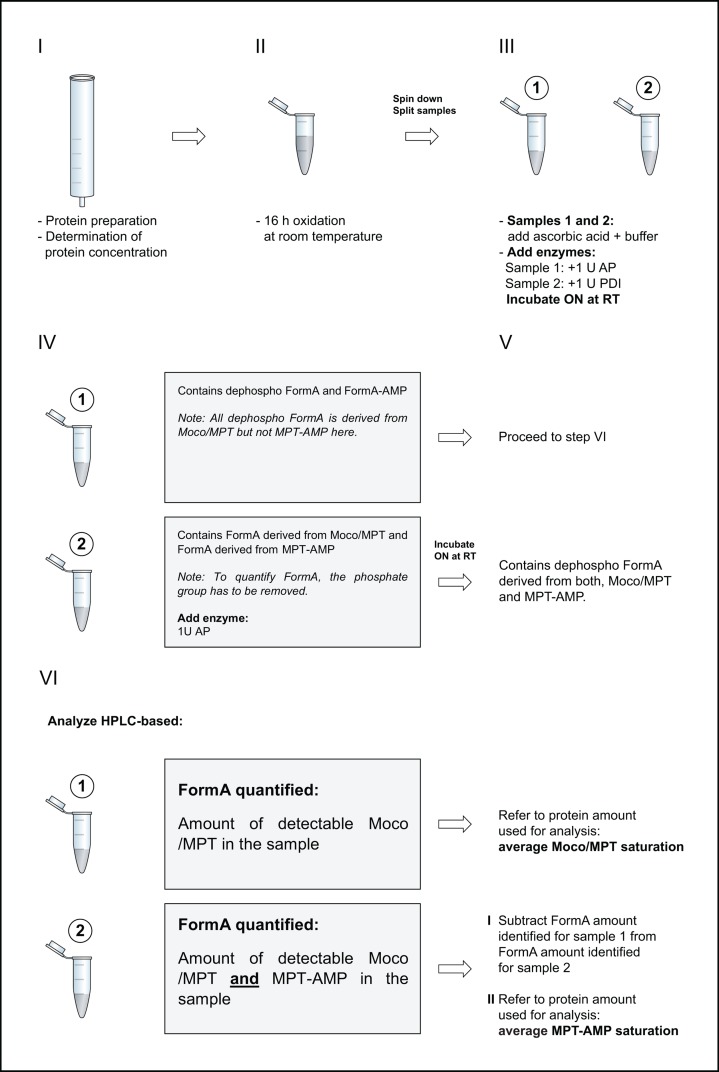
Protocol for FormA based Moco/MPT and MPT-AMP quantification FormA-based Moco/MPT and MPT-AMP quantification involves six steps. If feasible the protein preparation (step I) should be carried out under low oxygen conditions to prevent oxidative damage to Moco/MPT and/or MPT-AMP. Directly upon preparation, an aliquot of the recombinant protein is used for Moco/MPT and/or MPT-AMP quantification via FormA (step II). Oxidation is achieved by adding acidified I_2_/KI solution to the protein sample. In step III, the oxidized protein sample (containing FormA and FormA-AMP) is split. Excess iodine is reduced with ascorbic acid and the pH of the samples is adjusted to basic conditions suitable for subsequent enzymatic de-adenylation (phosphodiesterase I, PDI) or enzymatic dephosphorylation (alkaline phosphatase, AP). After overnight (ON) incubation, dephospho FormA derived from enzymatic dephosphorylation can be directly analyzed via HPLC, while FormA derived from the FormA-AMP de-adenylation (sample 2) is subjected to a dephosphorylation step by AP. FormA amounts derived from Moco/MPT (sample 1) or derived from both MPT-AMP and Moco/MPT (sample 2) are quantified. To determine the MPT-AMP amount within the sample, FormA quantified in sample 1 needs to be subtracted from FormA quantified in sample 2. Please note that variations in the FormA amounts quantified may be related to the effectiveness of Moco/MPT and/or MPT-AMP extraction from its protein environment during the oxidation procedure.

The protocol described here was adapted and modified from previous protocols [11,15,19,20] and used synthetic dephospho FormA [21] for Moco/MPT quantification in the biological samples. To quantify Cnx1E bound MPT-AMP and Moco/MPT, the protein preparations were processed directly after elution from the Strep-Tactin® Superflow® high-capacity resin (IBA) and prior to concentrating the sample thus ensuring minimal degradation of protein bound Moco/MPT and/or MPT-AMP, respectively. HPLC-based analysis first requires the conversion of Moco/MPT into the stable fluorescent derivative FormA [20] and of MPT-AMP into FormA-AMP [11,12], respectively. Therefore, 10–30 µl of the pooled elution fractions (containing ∼100 pmol protein) were added to pre-mixed oxidation preparations (each containing 800 µl 0.1 M Tris-HCl, pH 7.2 + 100 µl acidified 1% I_2_/ 2% KI solution). The I_2_/KI stock-solution was prepared as described [22] and directly before its use for Moco/MPT oxidation, HCl was added (final concentration = ∼1 M). The concentration of the protein solution was determined using the Bradford assay (Roti-Quant; Roth) with bovine serum albumin serving as a concentration standard [14]. Oxidation of the proteins was performed overnight for at least 16 h at 22°C. After oxidation, precipitants were removed by a centrifugation step (16,000 × *g*, 10 min, room temperature). Next, twice 450 µl of the supernatant was transferred into fresh reaction tubes yielding sample 1 and 2, respectively. Residual iodine was reduced by the addition of 50 µl of an 1% w/v aqueous ascorbic acid solution to both samples. Subsequently, 200 µl of a 1 M Tris solution and 13 µl 1 M MgCl_2_ were added to each sample. For quantification of FormA, 1 U of alkaline phosphatase (New England Biolabs) was added to sample 1. For quantification of FormA-AMP, 1 U of phosphodiesterase I (MP Biomedicals) was added to sample 2. Both samples were incubated at room-temperature for at least 16 h. Afterward, the alkaline phosphatase treated sample 1 was ready for analysis. To sample 2 (processed with phosphodiesterase I), 1 U alkaline phosphatase was added to convert FormA – resulting from FormA-AMP deadenylation – into dephospho FormA suitable for HPLC-based FormA quantification. After another overnight incubation step (at least 16 h) at room temperature, sample 2 was also ready for analysis (Figure 1 illustrates the protocol described here). HPLC analysis was carried out at room temperature using a reversed phase C-18 column (250 mm × 4.6 mm, 5 µm, ReproSil-Pur Basic C-18 HD) and an Agilent 1100 system consisting of a binary pump, autosampler and fluorescence detector. Dephospho FormA was eluted at a flow rate of 1 ml min^−1^ using an isocratic mobile phase containing 5 mM ammonium acetate and 15% (v/v) methanol, and had a specific retention time of 5.25 min. Dephospho FormA was detected fluorometrically (*λ* ex = 302 nm, *λ* em = 451 nm). All data were collected and processed with OpenLab CDS Version 2.2.0.600. Calibration was carried out using synthetic dephospho FormA [21] as calibration standard. For serial dilution of synthetic dephospho FormA, the protein-free FormA preparation-buffer (see above) was used.

### Cnx1E MPT-AMP hydrolysis assay

The Cnx1E MPT-AMP hydrolysis assay was essentially performed as described earlier [14]. MPT-AMP and Moco/MPT were quantified as described above.

### Inductively coupled plasma mass spectrometry

Quantification of the Cnx1E molybdenum content was carried out using an Agilent 7700 Series inductively coupled plasma mass spectrometry (ICP-MS; Agilent Technologies). For calibration, a standard curve of sodium molybdate (1–20 mg/l, Fluka) was used. Protein solutions and standards were mixed automatically using rhodium (Rh(NO_3_)_3_) as an internal standard. All values were corrected for the molybdenum background of control samples (buffer without protein). Data collection and processing were carried out using the MassHunter work station software.

### Crystallization, data collection and model building

Prior to crystallization, the protein was concentrated to about 30 g/l using Vivaspin concentrator columns with a molecular weight cut-off of 30 kDa. Concentrated protein solutions were supplemented with 0.015 M of both MgCl_2_ and ADP. Best crystals of Cnx1E variant D274E were obtained from various conditions of the Morpheus screen (Molecular dimensions). The crystals were flash-cooled in liquid nitrogen and subjected to X-ray diffraction experiments on beamline P11, operated by DESY at the PETRA III synchrotron (Hamburg, Germany) [23]. The data set was processed with autoPROC [24] and corrected for anisotropy with STARANISO [25]. The crystallographic phase problem was solved with Phaser [26] by transplanting phases from the previously determined Cnx1E wild-type structure (PDB entry: 6ETD, [15]). The initial structure was improved by refinement with Buster 2.10.3 [27] and rebuilding in Coot [28]. Amino acids with disordered side chains were modeled as stubs extending only to the β-carbon atom. In contrast, amino acids with a disordered backbone were not at all included in the structural model and are reflected by chain breaks. To avoid clashes between crystallographic neighbors near a special position, D435 and I436 were modeled as stubs despite interpretable side chain density. During the refinement, the atomic displacement factors were treated as being isotropic and domain displacement was accounted for by modeling two rigid body domains undergoing translation/libration/screw vibrational motion. The refinement was stopped after *R*_work_ and *R*_free_ converged. The files containing the structure factors and the structural model were deposited with the Protein Data Bank with accession number 6RMS. The complete data collection and refinement statistics are shown in Table 1.

**Table 1:** Data collection and refinement statistics

	Cnx1E-D274E
Wavelength (Å)	0.9537
Space group	I222
Unit cell parameters	
*a* (Å)	64.84 ± 0.04
*b* (Å)	119.48 ± 0.06
*c* (Å)	137.87 ± 0.06
α≡β≡γ (°)	:= 90
Resolution (Å)	
*d*_hkl,max_ – *d*_hkl,min_	90.01–1.74 (1.81–1.74)
*d*_h00,eff_	1.74
*d*_0k0,eff_	2.53
*d*_00l,eff_	1.85
*d*_eff,mean_^1^ [*d*_opt_]	1.99 [∼1.8]
No. of reflections	
Total	457,830 (2195)
Unique	34,684 (166)
Completeness	
Spherical	0.667 (0.160)
Ellipsoidal^2^	0.927 (0.654)
Multiplicity	13.2 (13.03)
Mean *I/σ(I)*	25.6 (1.5)
Wilson B (Å^2^)	31.5
*R*_merge_	0.058 (1.663)
*R*_meas_	0.060 (1.731)
*R*_pim_	0.016 (0.475)
CC1/2	1.000 (0.639)
No. of reflections used	34,673 (165)
*R*_work_ / *R*_free_	0.1983 / 0.2248
No. of non-hydrogen atoms	
Total	3217
in protein	2974
in ligands	15
in ordered solvent	228
Atomic *B*-factors (Å^2^)	
Average	43.4
Protein/Ligands/Solvent	43.3 / 61.4 / 43.3
No. of amino acid residues	
total / ordered	470 / 397
RMSD from ideal	
bonds (Å)	0.014
angles (°)	1.65
Ramachandran (%)	
favored	97.92
allowed	1.82
outliers	0.26

Numbers in parentheses account for the shell of highest resolution. ^1^Effective (*d*_eff_) and corresponding optical (*d*_opt_) resolution of the dataset determined with EFRESOL [33]. ^2^Data completeness for a volume in reciprocal space bounded by an ellipsoid centered on {000} and with the dimensions *a* = 1/*d*_h00,min_, *b* = 1/*d*_0k0,min_, *c* = 1/*d*_00l,min_.

## Results

### Cnx1E catalyzed MPT-AMP hydrolysis

**Figure 2:**
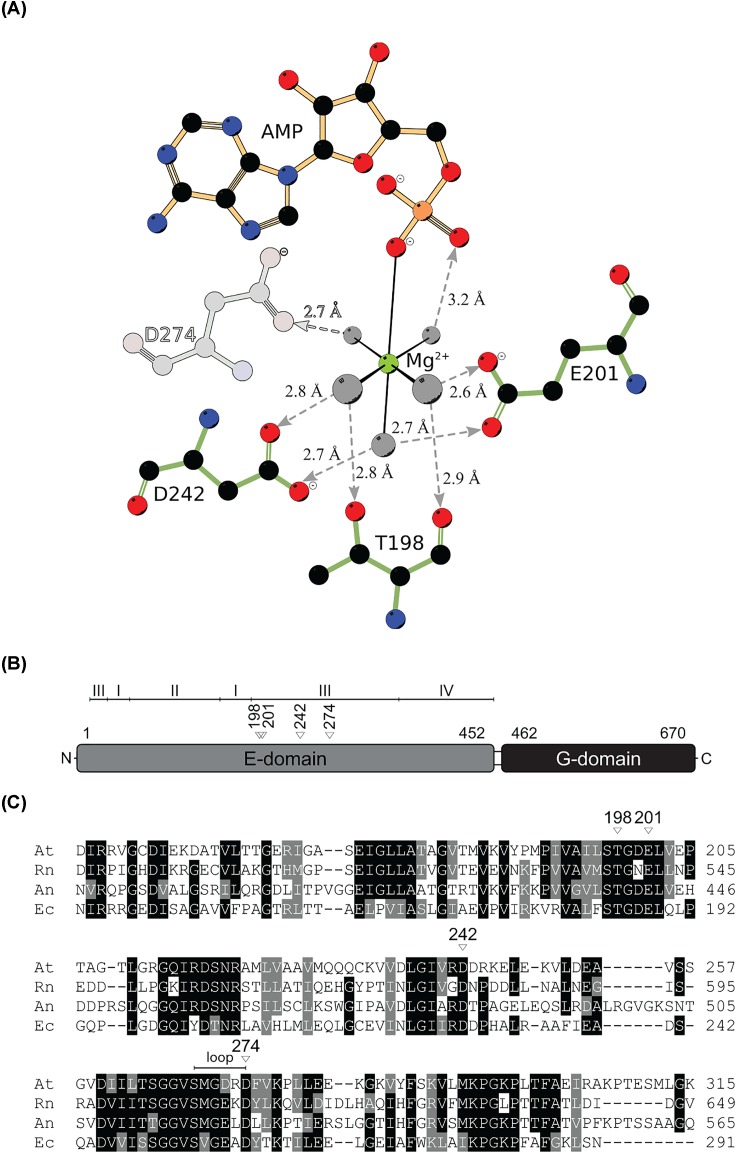
Cnx1E magnesium coordinating residues (**A**) Mg^2+^-AMP interacting amino acid residues are shown in simple stick representation and numbered. Protein to Mg^2+^-AMP interactions of Cnx1E variant D274E are identical with the exception of the missing interaction to residue E274. The atoms of residue D274 were shown semi-transparent to illustrate this. The Cnx1E bound AMP molecule is shown in ball-and-stick representation. The single co-crystallized magnesium ion is shown as green sphere, coordinated water molecules as gray spheres. Hydrogen bonds are represented by dashed, gray lines. If discernible, arrowheads point to the hydrogen bond acceptor with corresponding distances given in Ångströms (Å). (**B**) Schematic representation of the *Arabidopsis thaliana* Cnx1 enzyme domain structure [14,15]. The first and last residues of the domains are indicated. For Cnx1E subdomains I to IV are indicated. Residues involved in directed Mg^2+^ interactions are given above the Cnx1E domain structure. (**C**) Partial sequence comparison of *Arabidopsis thaliana* (At), *Rattus norvegicus* (Rn), *Aspergillus nidulans* (An) and *Escherichia coli* (Ec) Cnx1E homologs. Cnx1 residues involved in directed Mg^2+^ interactions are indicated by white triangles with the corresponding Cnx1 amino acid positions given above. Strictly conserved residues are highlighted in black, conserved residues are highlighted in gray. The alignment was generated with Clustal Omega. Figure part B and the corresponding caption was originally published in [15] (https://portlandpress.com/biochemj/article/475/10/1739/49736/) and has been modified here.

We recently reported a high-resolution Cnx1E structure in complex with active site bound Mg^2+^-AMP and molybdate [15]. However, the co-crystallization of the Cnx1E enzyme substrate complex was not possible, thus excluding the structure assisted elucidation of Cnx1E reactivity. Previously, the *in vitro* transfer of MPT-AMP on the recombinant Cnx1E wild-type enzyme was reported [13,14]. Using these methods, the routine production of Cnx1E with MPT-AMP occupancies sufficient for co-crystallization experiments was not successful, which could be due to Cnx1E- MPT-AMP hydrolysis activity retained in the crystallization conditions. We identified Cnx1E active site residues Thr198, Glu201, Asp242 and Asp274 as potential targets for site-directed mutagenesis to abolish MPT-AMP hydrolysis activity. These residues are conserved among Mo-insertases from various species (Figure 2C) and were shown to interact with the Mg^2+^ ion water shell molecules (Figure 2A, [15]) in the previously published Cnx1E structure [15]. They appear to be crucial for the proper positioning of the Mg^2+^ ion within the active site and may play a role in the activation of a water molecule for the nucleophilic attack of the MPT-AMP phosphate-phosphate bond [13]. In *Aspergillus nidulans*, the exchange of the *Arabidopsis thaliana* Asp274 corresponding residue (Asp522) for a glutamate residue results in a complete loss of Mo-insertase functionality [29]. We proceeded to characterize the corresponding Cnx1E exchange variant (D274E).

### Quantitative analysis of Cnx1E variant D274E

**Figure 3:**
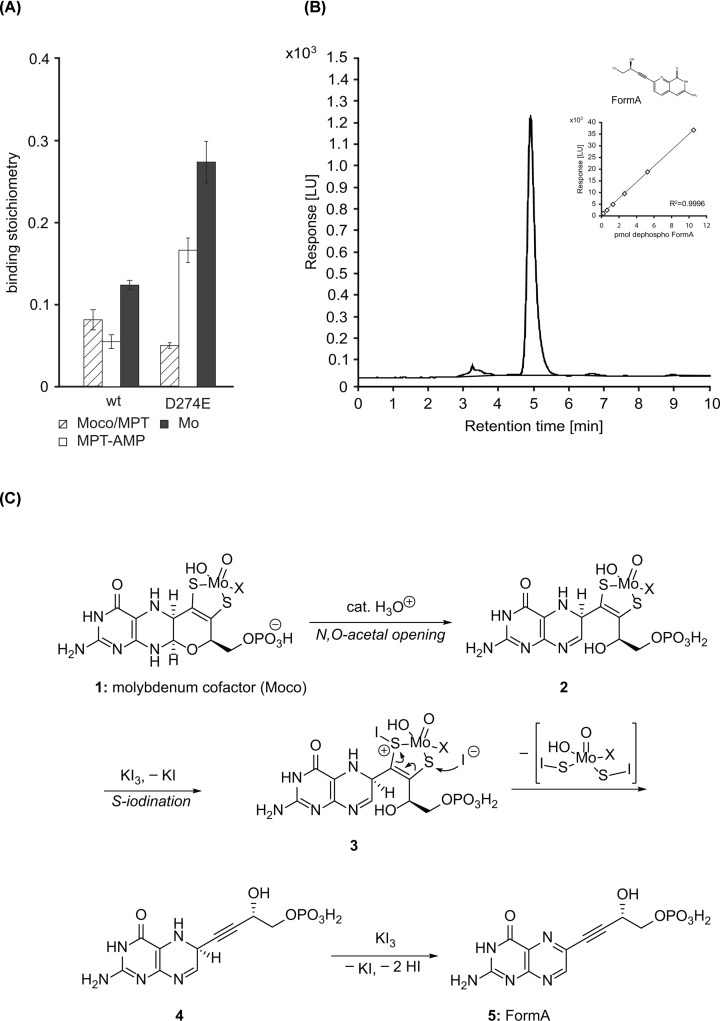
Biochemical characterization of CNX1E variant D274E (**A**) Recombinant Cnx1E wild-type (wt) protein and variant D274E were analyzed for co-purified molybdenum cofactor (Moco) / molybdopterin (MPT), Mo and adenylated MPT (MPT-AMP) after expression and purification from *E. coli* strain RK5206 [30]. Analysis of the recombinant protein was carried out as described in the ‘Materials and Methods’ section. Bars represent the standard deviation, resulting from three full replicas. (**B**) HPLC elution profile of 5.258 pmol synthetic dephospho FormA. A representative calibration curve used for quantitative analysis shown in panel (B) is shown as a second inset beneath the chemical structure of FormA (Y = PO_3_H^−^). For dephospho FormA Y = H, [21]. The lower detection limit was 0.328 pmol dephospho FormA. (**C**) Proposed mechanism of the conversion of Moco (1) to Form A (5). Formation of the triple bond of Form A requires prior opening of the dihydropyran ring present in Moco (1), because otherwise high strain energy would arise. Thus, as the first step, acidic hydrolysis of the N,O-acetal moiety of Moco must happen first, possibly affording bicyclic dihydropterin 2. Formally, two equivalents of molecular iodine are required to oxidize 1 to Form A (5). Given the high affinity of iodine to sulfur, it is proposed that one of the two sulfur atoms is iodinated electrophilically, as it has been observed for molybdenum sulfur complexes [34]. The liberated, nucleophilic iodide could attack the second sulfur atom (structure 3). Elimination of, probably unstable, diiodinated dithiomolybdate would generate the alkyne moiety of Form A. In the final step, a second equivalent of iodine may be consumed for the oxidation of dihydropterin 4 to pterin 5. Throughout the sequence, Mo(VI) would have kept its oxidation state. Oxidative desulfuration and dehydrogenation to the pterin unit might also occur in the reversed order. When starting from MPT, which lacks molybdenum, a similar pathway would lead to the formation of two molecules of HSI, which would be equilibrium with other iodinated sulfur species [35,36]. Next to FormA also FormB was described as stable, fluorescent Moco/MPT derivative [22]. In the absence of KI_3_, boiling of Moco-containing enzymes at pH 7 in the presence of 0.01 M Tris-HCl has led predominantly to Form B (11, Supplementary Figure S3). Formation of the thiophene ring of 11 requires rotation around the double bond, presumably via the thione tautomer 6 that is formed after hydrolysis of the Mo-S-bond and tautomerization. A first proposed mechanism for its formation is given in Supplementary Figure S3.

To give insights into the impact of residue Asp274 for MPT-AMP hydrolysis, we initially expressed and purified Cnx1E variant D274E from *E. coli* strain RK5206 [30]. Wild-type Cnx1E from RK5206 [14] served as a reference in the subsequently carried out biochemical comparison. Protein purities of wild-type Cnx1E and Cnx1E variant D274E were found to be equivalent (Supplementary Figure S1), thus allowing their direct comparison with respect to molybdate / MPT-AMP binding and hydrolysis activity. For Moco, metabolite quantification synthetic dephospho FormA [21] (Figure 3B) may be used as calibration standard for HPLC-based FormA quantification, representing a suitable alternative to the calibration method described earlier [19,20]. Having hands on synthetic dephospho FormA for the HPLC calibration likewise allowed us to establish faster FormA sample preparation protocols (Figures 1 and 3C, see ‘Materials and Methods’ section for details). FormA based HPLC analysis revealed Cnx1E variant D274E to accumulate MPT-AMP (0.17 +/- 0.03 MPT-AMP per monomer), while wild-type Cnx1E was found to be co-purified with significantly less MPT-AMP (0.06 +/- 0.02 molecules per monomer, Figure 3A). Next to MPT-AMP also Moco/MPT was found to be co-purified with both proteins (D274E = 0.05 +/- 0.01 molecules per monomer, wild-type Cnx1E = 0.08 +/- 0.03 molecules per monomer, Figure 3A). Since the quantified Mo-amount was 0.27 +/- 0.05 (D274E, Figure 3A), we presume that exclusively Moco but not MPT was co-purified with Cnx1E variant D274E. Since MPT-AMP and molybdate are bound equimolar by Cnx1E [13], we deduce that next to Mo bound in Moco, extant Mo quantified by ICP-MS originates from enzyme bound molybdate awaiting hydrolysis driven Mo-insertion into MPT-AMP. For wild-type Cnx1E, the total Mo-amount quantified was 0.12 +/- 0.01 (Figure 3A) suggesting that next to Moco and MPT-AMP + molybdate, minor amounts of MPT were co-purified here.

### *In vitro* Moco synthesis

**Figure 4:**
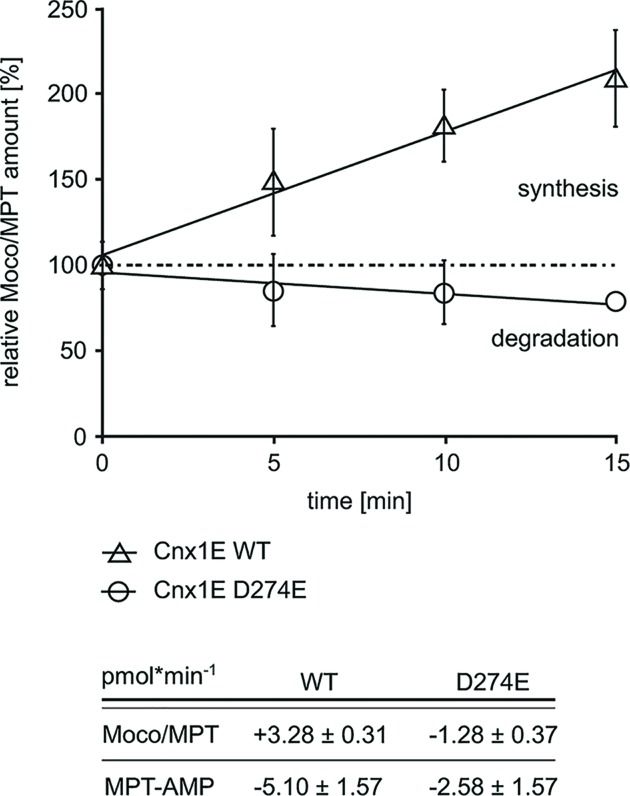
Moco synthesis activities of wild-type Cnx1E and Cnx1E variant D274E (Upper part) Moco formation of Cnx1E wild-type (wt) and variant D274E (see the ‘Materials and Methods’ section for details). For the sake of comparison, the Moco/MPT amounts were normalized to 100% (Cnx1E wt = 45.26 +/- 2.64 pmol; Cnx1E variant D274E = 92.07 +/- 13.45 pmol). Bars represent the standard deviation, resulting from five full replicas. The linear regression fit for both reactions is shown. Lower part: Wild-type Cnx1E catalyzed Moco formation occurs with 3.28 +/- 0.31 pmol Moco per min, whereas Moco/MPT degradation was observed for Cnx1E variant D274E (1.28 +/- 0.37 pmol Moco/MPT per min).

Next to the quantification of enzyme bound MPT-AMP and molybdate, we tested Cnx1E variant D274E for its ability to hydrolyze enzyme bound MPT-AMP. The substrate bound enzymes analyzed were obtained upon *in vitro* MPT-AMP loading [14]. A fully defined *in vitro* system was then employed to document the impaired MPT-AMP hydrolysis capacity of Cnx1E variant D274E (Figure 4). To do so, prior to the experiment, the Cnx1E wild-type and D274E concentrations were adjusted to ∼16% MPT-AMP saturation. Cnx1E variant D274E shows no Moco synthesis capacity (Figure 4). However, we documented Moco/MPT degradation here (-1.28 +/- 0.37 pmol Moco/MPT per min) that we attribute to oxidative damage of Moco/MPT. This also explains MPT-AMP consumption (Figure 4) of the demonstratively inactive (this work and [29]) Cnx1E variant D274E. To shed light on the molecular function of Cnx1E residue Asp274 with respect to magnesium ion coordination (Figure 2A), we proceeded to solve the structure of the Cnx1E D274E variant.

### The structure of Cnx1E variant D274E

**Figure 5:**
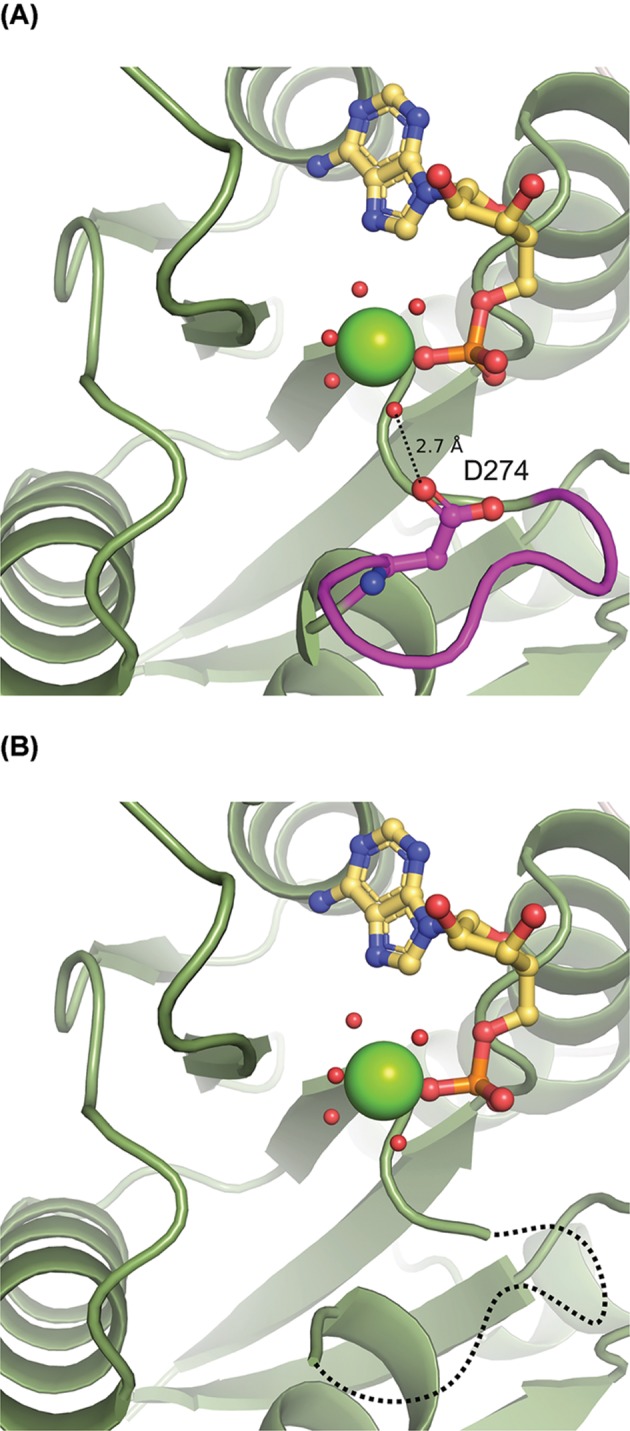
Close-up on the Cnx1E active site (**A**) Wild-type Cnx1E with Asp274 interacting with the water shell of Mg^2+^. (**B**) Cnx1E variant D274E with the disordered loop region indicated by a broken line. Residue 274 could not be located in the electron density due to disorder.

The overall structure of Cnx1E variant D274E resembles the Cnx1E wild-type structure published recently [15]. As a notable difference, no interpretable electron density is visible in the variant structure for the region comprising amino acids 269 through 274. However, the active site magnesium ion [15] was found to be in place (Figure 5). The disordered region 269 to 274 was confirmed to be highly dynamic in D274E through ensemble refinement [31]. In the wild-type structure, residue Asp274 interacts with the octahedral water shell of the catalytically relevant magnesium ion and is therefore involved in its coordination (Figures 2A and 5A). Replacement of Asp274 with glutamate seems to prevent this interaction and to give way to reorientation and disorder of that region (Figure 5B).

## Discussion

**Figure 6:**
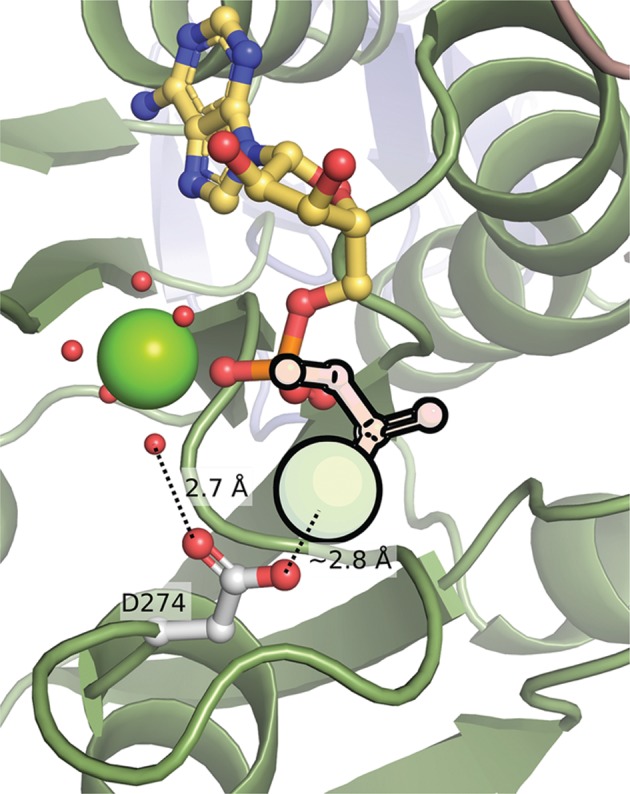
Cnx1E wild-type active site superimposed with a second Mg^2+^ present in the active site of GephE The Mg^2+^ and the β-phosphate group of the ADP molecule from the GephE structure (PDB entry: 5ERR) are shown in pale with black outline. Asp274 from Cnx1E is the positional equivalent to Asp580 in GephE, which directly interacts with the second Mg^2+^ and with the water shell of the first.

In this work, we describe the biochemical and structural characterization of the Cnx1E variant D274E. Both Cnx1E substrates, i.e. MPT-AMP and molybdate were co-purified with the recombinant protein in approximately equimolar quantities that is in line with the previous finding that Cnx1E binds MPT-AMP and molybdate cooperatively [13]. Recombinant biochemistry revealed Cnx1E variant D274E to be hydrolysis inactive, thus we conclude that missing hydrolysis activity causes both MPT-AMP and molybdate to be arrested at the active site. We identified a disordered active site loop (spanning amino acids Ser269 to Asp274) as a potential reason for this hydrolysis inactivity of Cnx1E variant D274E. In Cnx1E wild-type, this active site loop partially covers the putative MPT-AMP binding site [14,15]. However, as the MPT-AMP binding properties of Cnx1E variant D274E were found to be wildtype-like, we conclude that functionality of residue Asp274 is restrained to the Mg^2+^ dependent MPT-AMP hydrolysis mechanism [13]. Notably, in the human Cnx1E homolog, GephE, a second cation void of an octahedral water shell is found in the active site ([32], Supplementary Figure S2). This cation is only seen when ADP is co-crystallized with GephE, as it is coordinated by both ADP phosphate groups. Next to these, only Asp580 of the GephE active site is involved in its coordination. Interestingly, Asp580 is the positional homolog to Cnx1E Asp274 (Figure 6), the amino acid we targeted by mutagenesis in this work and given that Moco biosynthesis is an evolutionary old and strictly conserved pathway [1,2], it cannot be excluded that also Cnx1E catalyzed MPT-AMP hydrolysis [13] relies on a second cation.

This notion is supported by the fact that Cnx1E Asp274 belongs to a set of active site residues that are strictly conserved among various eukaryotic Mo-insertases [15]. However, all attempts to co-crystallize Cnx1E with ADP failed, as ADP was not stable under the crystallization conditions (this work and [14,15]) and hence the presence of the (ADP coordinated) second active site cation cannot be confirmed for Cnx1E. We showed, that the catalytically important Asp274 residue interacts with a solvent molecule of the Mg^2+^ water shell. As the Cnx1E D274E variant is hydrolysis inactive, we conclude that the interaction of Asp274 with said Mg^2+^ water shell molecule is essential for MPT-AMP hydrolysis.

## Supplementary Material

Supplementary Figures S1-S3

## Abbreviations

AP: alkaline phosphatase
cPMP: cyclic pyranopterin monophosphate
ICP-MS: inductively coupled plasma mass spectrometry
Moco: molybdenum cofactor
Mo-insertase: molybdenum insertase
MPT: molybdopterin
MPT-AMP: adenylated MPT
PDI: phosphodiesterase I
