# Supplementary material for: Insights into the Cnx1E catalyzed MPT-AMP hydrolysis
Source: Biosci Rep. 2020 Jan 10;40(1):BSR20191806. doi: 10.1042/BSR20191806 (PMC6954367; doi:10.1042/BSR20191806)
Supplement: Supplementary Figures S1-S3 [file BSR-2019-1806_supp.pdf]

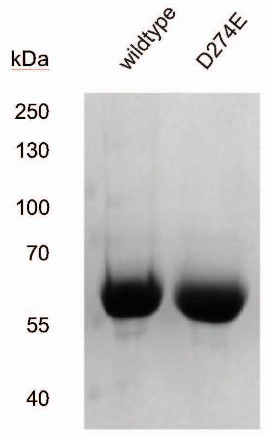

Figure -S1-

Figure S1: Biochemical characterization of CNX1E variant D274E. (A) Representative SDS-polyacrylamide gel displaying the purity of Cnx1E wildtype and variant D274E protein preparations.

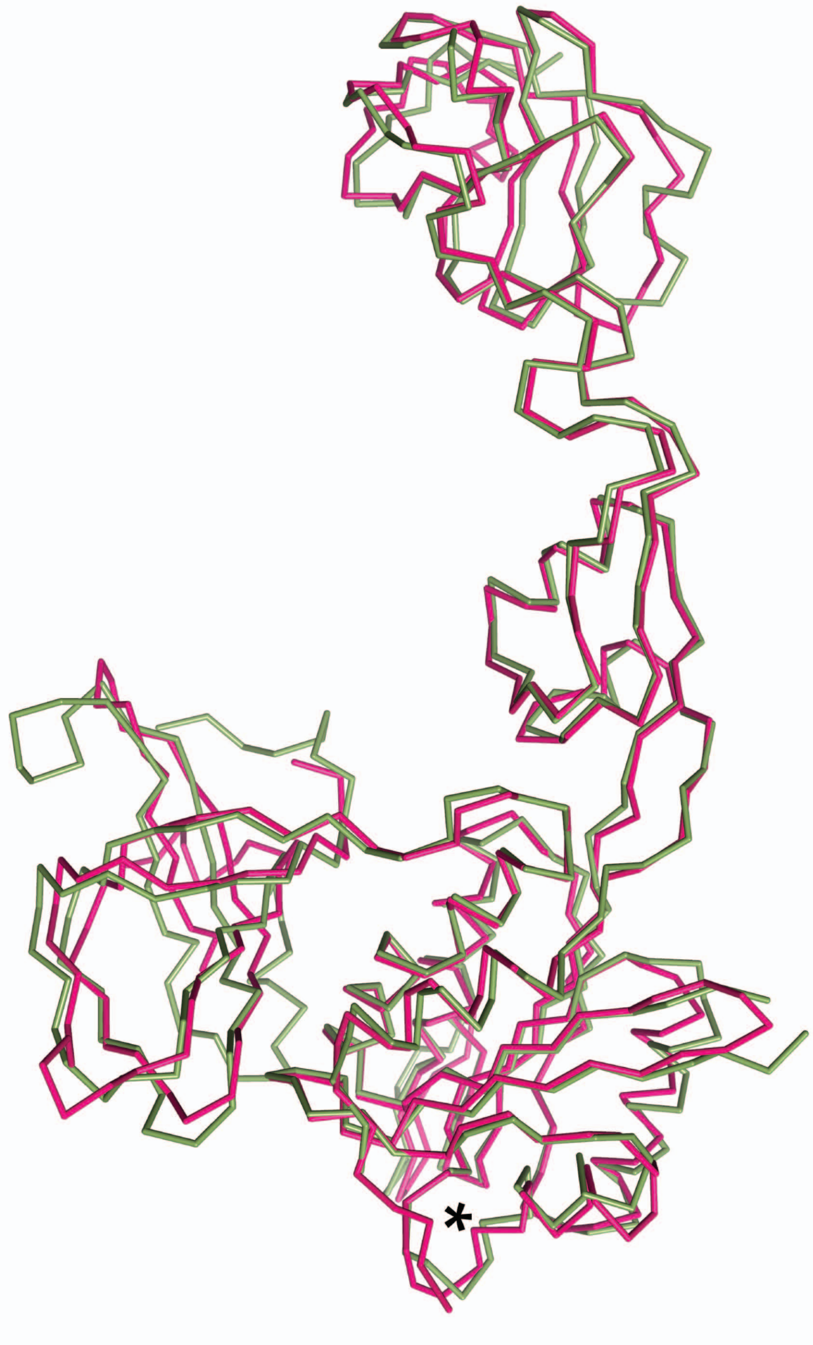

Figure -S2-

Figure S2:Comparison of Mo-insertase structures. Cnx1E from *Arabidopsis thaliana* (green) and GephE *Rattus norvegicus* (pink) were superimposed with an C-alpha R.M.S.D of 1.014 Å for the 347 best aligning amino acid pairs and of 2.789 Å for all pairs. The asterisk indicates the location of Asp274 and Asp580 and Cnx1E and GephE, respectively.

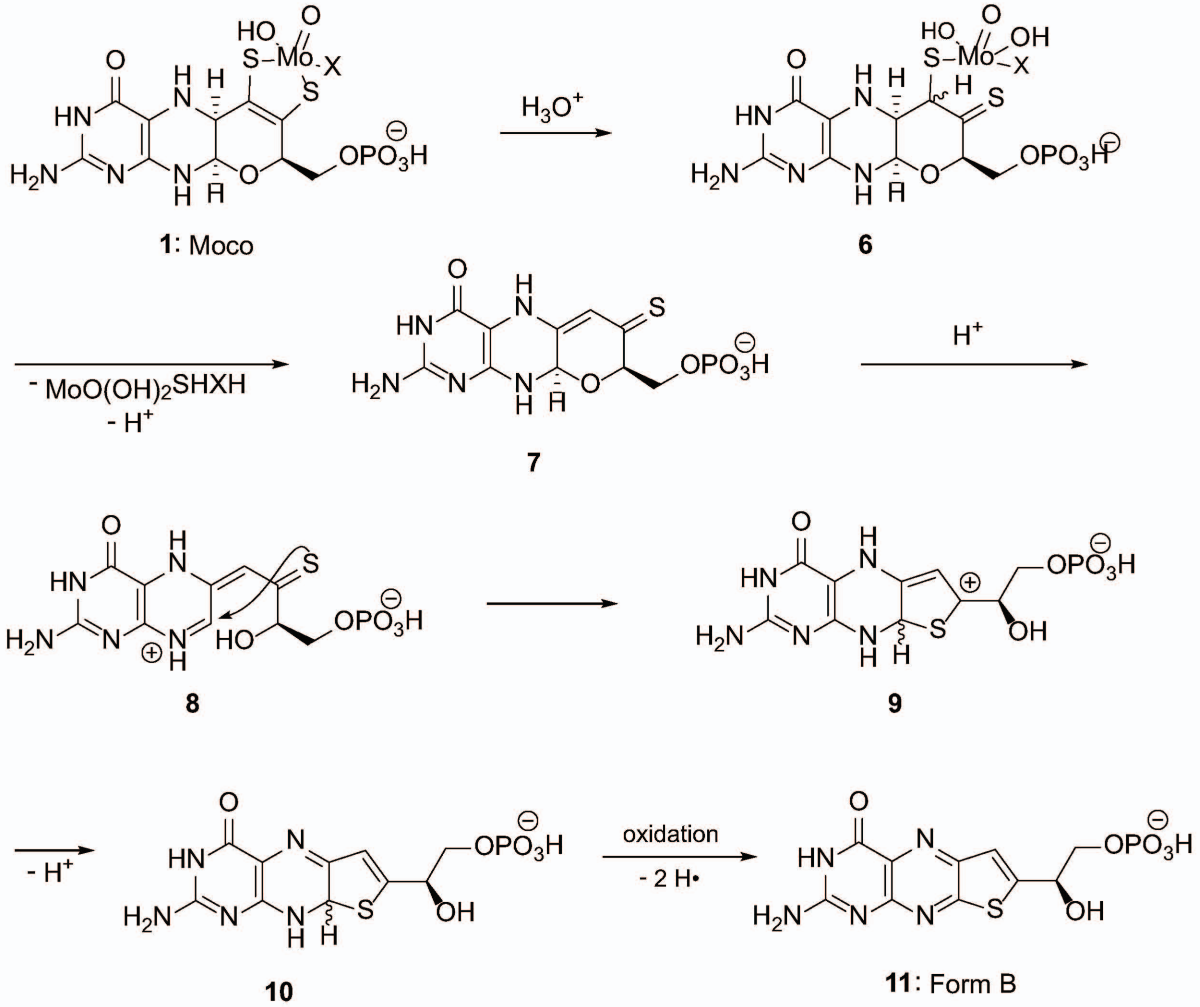

Figure -S3-

Figure S3: Proposed mechanism of the conversion of Moco (1) to Form B (11). Differing from the formation of Form A (**5**), which is proposed to start with the iodination of the sulfur atoms (Fig. 3B), hydrolysis of the thiomolybdate unit to thione **6** could be the fastest step in the absence of iodine. Elimination of the Mo(VI) moiety could afford the α,β-unsaturated thioketone **7**, which would undergo N,O-acetal opening to intermediate 8. The nucleophilic thione sulfur would attack at the generated iminium ion **8** and thereby replace the oxygen. Deprotonation of the allylic cation **9** would lead to compound **10** that subsequently would undergo air oxidation to Form B (**11**).
